# IL-37 overexpression promotes endometrial regenerative cell-mediated inhibition of cardiac allograft rejection

**DOI:** 10.1186/s13287-022-02982-1

**Published:** 2022-07-15

**Authors:** Hong Qin, Chenglu Sun, Yanglin Zhu, Yafei Qin, Shaohua Ren, Zhaobo Wang, Chuan Li, Xiang Li, Baoren Zhang, Jingpeng Hao, Guangming Li, Hongda Wang, Bo Shao, Jingyi Zhang, Hao Wang

**Affiliations:** 1 Department of General Surgery, Tianjin Medical University General Hospital, 154 Anshan Road, Heping District, Tianjin, 300052 China; 2 Tianjin General Surgery Institute, Tianjin, China; 3 School of Basic Medical Sciences, Tianjin Medical University, Tianjin, China; 4 Department of Anorectal Surgery, Tianjin Medical University Second Hospital, Tianjin, China

**Keywords:** Endometrial regenerative cells, Interleukin-37, Acute allograft rejection, Mice

## Abstract

**Background:**

Endometrial regenerative cells (ERCs) play an important role in attenuation of acute allograft rejection, while their effects are limited. IL-37, a newly discovered immunoregulatory cytokine of the IL-1 family, can regulate both innate and adaptive immunity. Whether IL-37 overexpression can enhance the therapeutic effects of ERCs in inhibition of acute cardiac allograft rejection remains unknown and will be explored in this study.

**Methods:**

C57BL/6 mice recipients receiving BALB/c mouse heterotopic heart allografts were randomly divided into the phosphate-buffered saline (untreated), ERC treated, negative lentiviral control ERC (NC-ERC) treated, and IL-37 overexpressing ERC (IL-37-ERC) treated groups. Graft pathological changes were assessed by H&E staining. The intra-graft cell infiltration and splenic immune cell populations were analyzed by immunohistochemistry and flow cytometry, respectively. The stimulatory property of recipient DCs was tested by an MLR assay. Furthermore, serum cytokine profiles of recipients were measured by ELISA assay.

**Results:**

Mice treated with IL-37-ERCs achieved significantly prolonged allograft survival compared with the ERC-treated group. Compared with all the other control groups, IL-37-ERC-treated group showed mitigated inflammatory response, a significant increase in tolerogenic dendritic cells (Tol-DCs), regulatory T cells (Tregs) in the grafts and spleens, while a reduction of Th1 and Th17 cell population. Additionally, there was a significant upregulation of immunoregulatory IL-10, while a reduction of IFN-γ, IL-17A, IL-12 was detected in the sera of IL-37-ERC-treated recipients.

**Conclusion:**

IL-37 overexpression can promote the therapeutic effects of ERCs to inhibit acute allograft rejection and further prolong graft survival. This study suggests that gene-modified ERCs overexpressing IL-37 may pave the way for novel therapeutic options in the field of transplantation.

## Introduction

Organ transplantation is the most effective strategy for patients with end-stage organ failure of varying etiologies. Unfortunately, though in 2019 alone, 40,623 solid organ transplants were performed in the USA, and the waiting list continues to outpace the number of transplants performed [1]. Worse still, acute rejection is a common complication post-transplantation that strongly weakens the function of the allografts and threatens the patients' survival [2]. Current immunosuppression therapies have significantly decreased the incidence of acute allograft rejection; however, some deleterious adverse effects are along with that, such as drug toxicity [3], infections [4], metabolic complications [5], and malignancies [6]. Therefore, novel alternative therapeutic strategies are urgently needed to be developed.

With the great immunomodulatory and regenerative properties, mesenchymal stromal cells (MSCs) have generated growing enthusiasm as an innovative cell-based approach in immune-mediated diseases, including experimental colitis [7], systemic lupus erythematosus (SLE) [8, 9], as well as allograft rejection and so on [10]. MSCs possessing the potential to prolong graft survival has been demonstrated in preclinical animal transplant models of the kidney, heart, liver, and lung [11–14]. Moreover, phase I clinical trials have proved the efficacy, feasibility, and safety of MSCs treatment in kidney/liver transplantation [15–17]. Although bone marrow-derived mesenchymal stromal cells (BM-MSCs) have been predominantly studied, the characteristic of invasive acquiring procedure and limited proliferate rate are still the issues for the clinical use of BM-MSCs in large quantities [18].

Endometrial regenerative cells (ERCs), a novel kind of adult stem cells from human menstrual blood, have gradually become a promising alternative for cell-based therapy due to their comprehensive advantages, such as their rich source of materials, noninvasive acquiring procedure, superior proliferation capacity, and potential to be used for autologous transplantation [19–21]. Previous studies of our research group and others have demonstrated the outstanding therapeutic efficacy of ERCs in liver disease [22–25], myocardial infarction [26, 27], acute lung injury [28], inflammatory bowel disease [29, 30], allograft rejection [31–33], etc. Additionally, clinical trials using ERCs were reported in the treatment of multiple sclerosis [34], Duchenne muscular dystrophy [35], and congestive heart failure [36], and no adverse effects were reported in the follow-up reports. Therefore, ERCs could bring a bright future for cell-based therapy in the clinic.

Interleukin 37 (IL-37), a recently characterized member of the IL-1 family, plays a key role in limiting excessive inflammatory responses as a fundamental inhibitor of innate and adaptive immunity [37–39]. It has been demonstrated that the transgenic mice expressing human IL-37 gene can protect them from different pro-inflammatory situations, such as ulcerative colitis (UC) [40] and LPS-induced endotoxemia [38]. Moreover, we and others have reported that IL-37 has synergistic effects with MSCs or ERCs in attenuation of UC [41], intestinal ischemia–reperfusion injury [42], concanavalin A-induced hepatitis [43], and SLE [44] via different mechanisms. Recently, our ongoing study has also demonstrated the effects of recombinant IL-37 in inhibition of acute cardiac allograft rejection.

Hence, in this study, to further enhance the therapeutic effects of ERCs, we genetically modified ERCs with overexpression of IL-37 and determined whether IL-37 overexpressing ERCs could further protect cardiac allografts against rejection, thereby achieving long-term allograft acceptance.

## Materials and methods

### Isolation, expansion, and modification of ERCs

The isolation and expansion of ERCs were performed following the protocol as described previously [31, 32]. Briefly, ERCs were isolated from menstrual blood collected by healthy volunteers aged 20 to 40 years old using Ficoll density centrifugation. The procedure was under ethical approval from Tianjin Medical University General Hospital (Tianjin, China, IRB2020-YX-128-01). The isolated cells were then cultured in Dulbecco’s modified Eagle’s medium (DMEM, Hyclone, USA) supplemented with 10% FBS (CORNING, New Zealand), and 1% penicillin/streptomycin (Solarbio, Beijing, China). Every two or three days, the medium was changed to remove the tissue fragments and non-adherent cells. ERCs were passaged at 80% confluence.

To overexpress IL-37 (NM_014439, IL-37 isoform b) in ERCs, lentiviral transfection of ERCs was conducted according to the manual provided by GeneChem Inc., Shanghai, China. (The vector conducted as Ubi-MCS-3FLAG-SV40-GFP-IRES-puromycin and the vector lacking IL-37 gene were used as negative control.) Lentiviral transfection was done in a biosafety cabinet with an appropriate multiplicity of infection (MOI = 50). Three days post-transfection, the cells were observed under an inverted fluorescence microscope, and the transfection efficiency was assessed by visualizing green fluorescence protein (GFP) expression. After passage, ERCs were then screened with 2 μg/mL puromycin (Solarbio, Beijing, China) for 3 days. ERCs from Passage 5 were used for subsequent experiments. To ensure cell purity and quality, the generated IL-37-ERCs were tested by flow cytometry before treatment.

### Enzyme-linked immunosorbent assay (ELISA)

The levels of IL-37 in the cell culture medium were measured using an ELISA kit (4A Biotech, Beijing, China). The mouse IFN-γ, IL-17A, IL-12p70, and IL-10 ELISA kits purchased from DAKEWE (Beijing, China) were used to measure the cytokine levels in the sera. All experimental procedures were conducted according to the manufacturer’s handbooks.

### Animals

Age-matched BALB/C (H‐2^d^) and C57BL/6 (H‐2^b^) male mice (8–10 weeks, 24–26 g) were purchased from the China Food and Drug Inspection Institute (Beijing, China). All the experiments were approved by the Animal Ethical and Welfare Committee of Tianjin Medical University General Hospital (Tianjin, China, IRB2020-DW-19) and conducted according to the guideline of the Chinese Council on Animal Care.

For intra-abdominal heterotopic cardiac transplantation, BALB/c mice and C57BL/6 mice served as donors and recipients, respectively. The operation procedure has been described in our previous study [45]. After resuscitation, the recipient mice were randomly assigned to four groups (*n* = 6 per group), (1) untreated group; (2) ERC-treated group (naïve ERCs); (3) NC-ERC-treated group (ERCs transfected with lentiviral lacking IL-37 gene, used as negative control); and (4) IL-37-ERC-treated group (ERCs transfected with lentiviral carrying IL-37 gene). For treatment, 1 × 10^6^ cells per mice were administered intravenously 24 h before and after the surgery in ERC-, NC-ERC-, and IL-37-ERC-treated groups. For graft survival, beating of the transplanted heart was detected daily by abdominal palpation and recorded, and survival curves were depicted. For the assessment of efficacy and immune microenvironment, animals were euthanized at postoperative day (POD) 8, and the grafts and spleens were extracted for subsequent analysis.

### Isolation of mononuclear cells from grafts

The transplanted grafts were harvested at POD8 and placed in heparinized saline. After removal of epicardial adipose tissue, the heart was minced with a scalpel, and blood was flushed out by repeated washing in heparinized saline. The chopped tissue was digested with type II collagenase (2 mg/mL; Solarbio, Beijing, China), DNase I (100 μg/mL; Solarbio, Beijing, China), and trypsin (0.1%; Solarbio, Beijing, China) in 5 mL RPMI 1640 medium (Hyclone, USA) for 90 min at 37 °C with occasional shaking. Next, a 5 mL serum-containing medium was added to terminate the digestion. Then filtering the digests through a 70 μm cell strainer, collected cells were pooled and pelleted at 300 g for 5 min at 4 °C. The supernatant was discarded, and the cell pellet was then resuspended in 3 mL of 40% Percoll and carefully underlaid with 2 mL of 70% Percoll. After centrifuging, the middle white layer of mononuclear cells was collected for flow cytometry analysis.

### Flow cytometry analysis

ERCs were obtained and stained with surface marker (HLA-DR, CD79a, CD90, and CD105) fluorescent antibodies for phenotype identification. To evaluate the immune microenvironment of the recipients, splenocytes and mononuclear cells from the grafts were collected and performed by flow cytometry analysis. Before surface staining, cells were stained with Zombie NIR™ (BioLegend, USA) to identify dead/live cells. For surface staining, cells were incubated with appropriate antibodies for 50 min at 4℃. For intracellular staining, cells were washed after surface staining and then permeabilized and fixed using the fixation/permeabilization kit (Thermo Fisher Scientific). Next, the cells were washed twice and the cell pellets were resuspended in 100 μL of flow cytometry staining buffer (eBioscience, Thermo Fisher Scientific) and then stained with the surface staining procedure mentioned above. As for IFN-γ and IL-17A staining, the cell stimulation cocktail plus protein transport inhibitors (Thermo Fisher Scientific) were added and incubated for 6 h in a 37 °C, 5% CO_2_ incubator to stimulate cytokine production and prevent its secretion, followed by intracellular staining as described above. Antibodies used in the present study including HLA-DR-FITC (Clone: L243), CD79a-PE (Clone: HM47), CD90-PE (Clone: 5E10), CD105-PE-Cyanine7 (Clone: SN6), CD4-FITC (Clone: RM4-5), IFN-γ-PE (Clone: XMG1.2), IL-17A-Percp-cyanine5.5 (Clone: eBio17B7), CD25-PE (Clone: PC61.5), Foxp3-APC (Clone: FJK-16 s), CD11c-APC (Clone: N418), MHC-II-FITC (Clone: M5/114.15.2), and CD86-PE (Clone: GL1) were purchased from BioLegend or eBioscience.

### Histology

Grafts were harvested, cut longitudinally and fixed in 10% formalin for 48 h and then embedded in paraffin and sectioned at 5 μm intervals for hematoxylin and eosin (H&E) staining to assess the severity of rejection. Criteria for allograft rejection assessment included the presence of lymphocyte infiltration, vasculitis, infarction, myocyte necrosis, intravascular thrombosis, and interstitial hemorrhage [46]. To be specific, compared with normal tissue, those changes were scored as 0, no change; 1, minimum change, 2, mild change; 3, moderate change; and 4, marked change.

### Immunohistochemistry

To identify the intra-graft infiltration of CD4^+^ cells in different groups, immunohistochemistry was performed as previously described [43]. Briefly, 5-μm tissue sections were made from the paraffin-embedded grafts and incubated with 3% hydrogen peroxide to eliminate endogenous peroxides. Then, the sections were placed in a repair box filled with EDTA antigen repair buffer, and antigen repair was conducted for 15 min at 100 °C in the microwave oven. After blocking with 10% goat serum, the sections were incubated with rabbit anti-mouse CD4 antibody (dilution at 1:1000, Abcam) overnight at 4 °C and then incubated with 100 μL enhanced enzyme-labeled goat anti-rabbit IgG polymer (DAB kit, ZSGB-BIO, Beijing, China) for 20 min the next day at room temperature. Finally, the sections were stained with hematoxylin for 4 min. The proportion of CD4 positive area was recognized and calculated using Image J software.

### Mixed lymphocyte reaction (MLR)

Mixed lymphocyte reactions (MLRs) were performed to assess the ability of recipient DCs in the stimulation of allogeneic T cell proliferation. Specifically, CD11c^+^ DCs were purified from recipient C57BL/6 spleen cells from different groups by magnetic cell sorting (CD11c^+^, Miltenyi, Teterow, Germany). Then, the collected DCs (5 × 10^4^ cells/well) pretreated with mitomycin C (50 μg/mL, Solarbio, Beijing, China) served as stimulators, co-cultured with allogeneic CD4^+^ T cells (5 × 10^5^ cells/well) derived from BALB/c mice. After 96 h of co-culture, a cell counting kit‐8 (CCK‐8, Solarbio, Beijing, China) assay was used to assess the proliferation of lymphocytes by recording the absorbance at 450 nm on a microplate reader (Bio-Rad, USA).

### Statistics

All statistics were calculated with GraphPad Prism 7.0, and the collected experimental data were presented as mean ± SD. For the differences between multiple groups, one‐way analysis of variance (ANOVA) was used. The graft survival analysis was conducted by Kaplan–Meier cumulative survival method, and the survival differences among groups were analyzed by log‐rank (Mantel–Cox) test. *P < 0.05; **P < 0.01; ***P < 0.001.

## Results

### Characterization of ERCs and IL-37-ERCs

**Fig. 1:**
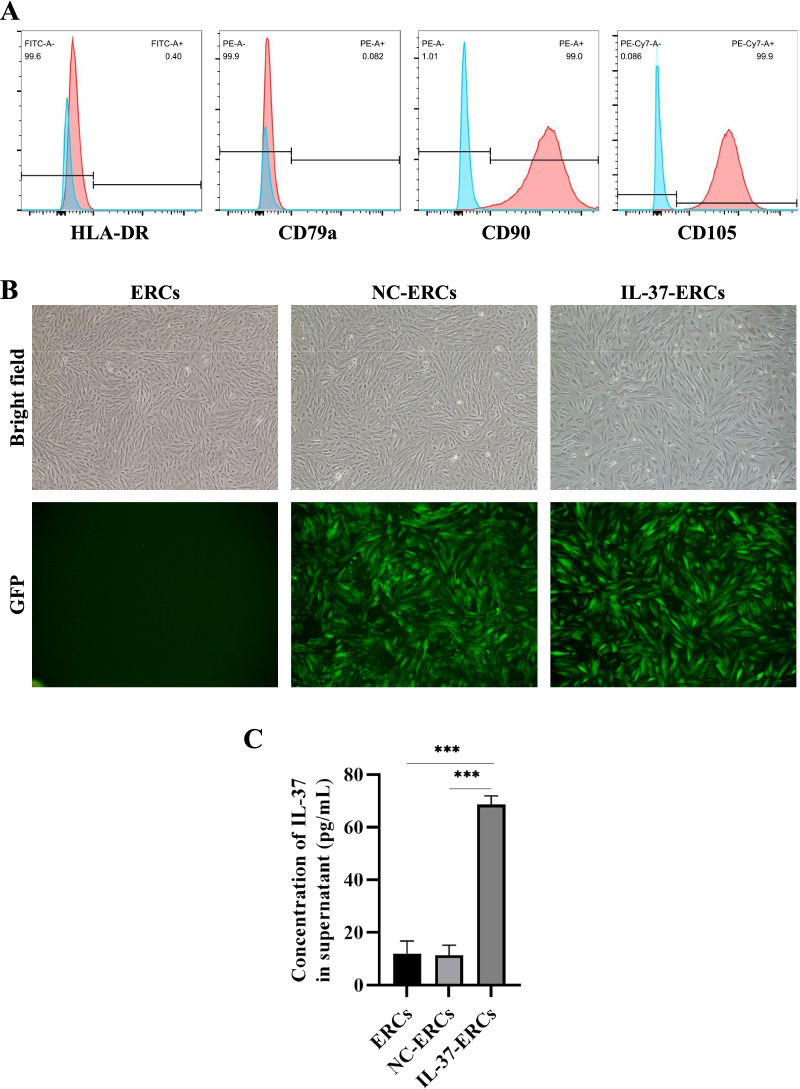
Identification of ERCs and IL-37-ERCs. **A** Flow cytometry analysis of surface markers of ERCs. **B** Morphology of ERCs, NC-ERCs, and IL-37 ERCs at passage 2 (magnification 40×). The successful transfection of ERCs with vector or IL-37 was shown by GFP. **C** Concentration of IL-37 in the culture supernatant of ERCs, NC-ERCs, and IL-37-ERCs. Statistical analysis was done by one-way ANOVA, ***P < 0.001

When ERCs grew to passage 3, morphology and surface markers were detected. ERCs exhibited a fibroblast-like phenotype and were positive for CD90, CD105, while negative for CD79a and HLA-DR (Fig. 1A, B). At 72 h post-transfection, the GFP expression can be detected in more than 80% IL-37-ERCs (Fig. 1B). In addition, successful overexpression of IL-37 was also confirmed by an ELISA assay (Fig. 1C).

### IL-37 overexpression promoted ERCs to prolong graft survival and alleviate acute allograft rejection

**Fig. 2:**
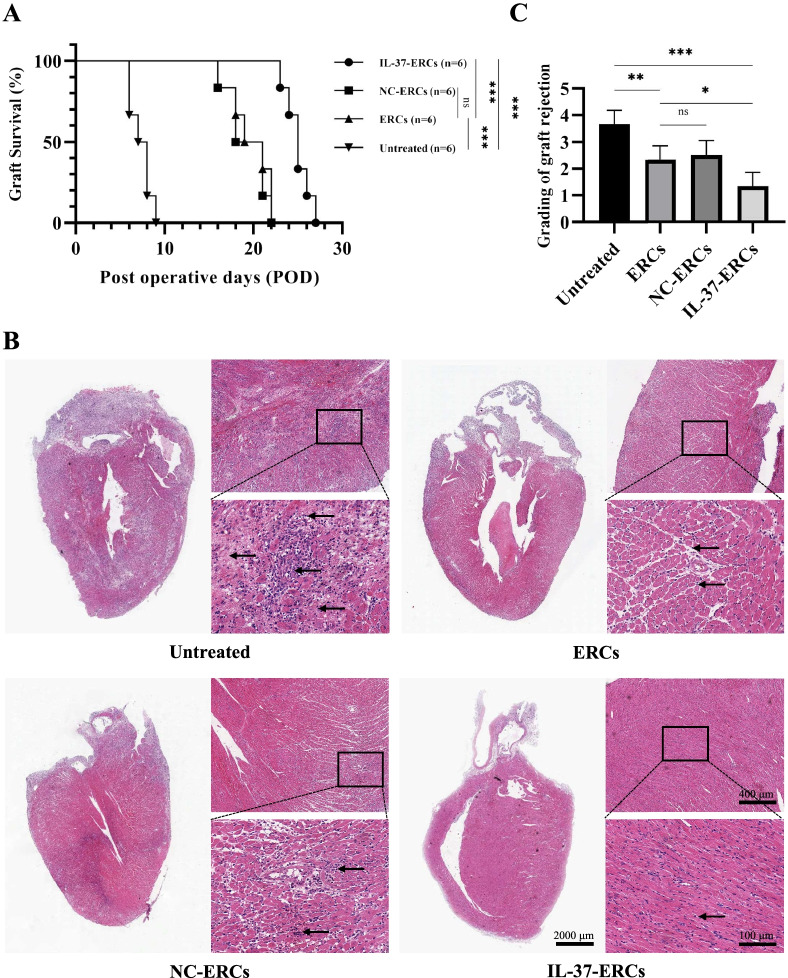
IL-37 overexpression promoted ERCs to prolong graft survival and alleviate acute allograft rejection. **A** Kaplan–Meier curves of allograft survival of fully MHC-mismatched cardiac transplants, in which BALB/c hearts were transplanted into C57BL/6 recipients. The allograft survival was significantly prolonged in the ERC group when compared with the untreated group, and further improved in the IL-37-ERC group (*n* = 6 per group). Statistics by Log-rank test. **B** Representative histology of cardiac allografts retrieved at the time of rejection. ERC-treated recipients revealed a milder lymphocytes infiltration and milder myocyte damage, while IL-37-ERCs further alleviated these changes (*n* = 6 per group). **C** Grading of graft rejection in different groups. Score criteria: 0, no change; 1, minimum change; 2, mild change; 3, moderate change; and 4, marked change compared with normal tissues (*n* = 6 per group). Statistics by one-way ANOVA. *P < 0.05, **P < 0.01, ***P < 0.001, ns = non-significant. For all panels, the bar graphs represent mean ± SD

To investigate whether IL-37 overexpression could promote ERC-mediated therapeutic effects on inhibition of allograft rejection, BALB/c (donor)-to-C57BL/6 (recipient) mouse intra-abdominal heterotopic heart transplantations were performed and administered respective treatments. The graft survivals were recorded, as shown in Fig. 2A, ERCs could prolong graft survival as compared with the untreated group (*P* < 0.001), and IL-37 overexpression further prolonged graft survival compared with ERCs (*P* < 0.001).

H&E staining in the grafts of untreated group showed typical features of acute rejection, characterized by vasculitis and massive inflammatory cell infiltration. As compared with the grafts of untreated group (3.667 ± 0.516), the severity of graft damage was alleviated in ERC-treated (2.333 ± 0.516, *P* < 0.01), NC-ERC-treated (2.500 ± 0.548, *P* < 0.01), and IL-37-ERC-treated (1.333 ± 0.516, *P* < 0.001) groups. Furthermore, the IL-37-ERCs showed an enhanced inhibitory effect on the allograft rejection compared with the ERCs (IL-37-ERC-treated group vs. ERC-treated group, 1.333 ± 0.516 vs. 2.333 ± 0.516, *P* < 0.05), while there were no significant differences between ERC-treated group and NC-ERC-treated group (ERC-treated group vs. NC-ERC-treated group, 2.333 ± 0.516 vs. 2.500 ± 0.548, *P* > 0.05), which means that negative control lentivirus may not influence the function of ERCs. These data implied that IL-37 overexpression could promote ERC-mediated inhibition of allograft rejection.

### IL-37-ERCs remodeled graft immune microenvironment

**Fig. 3:**
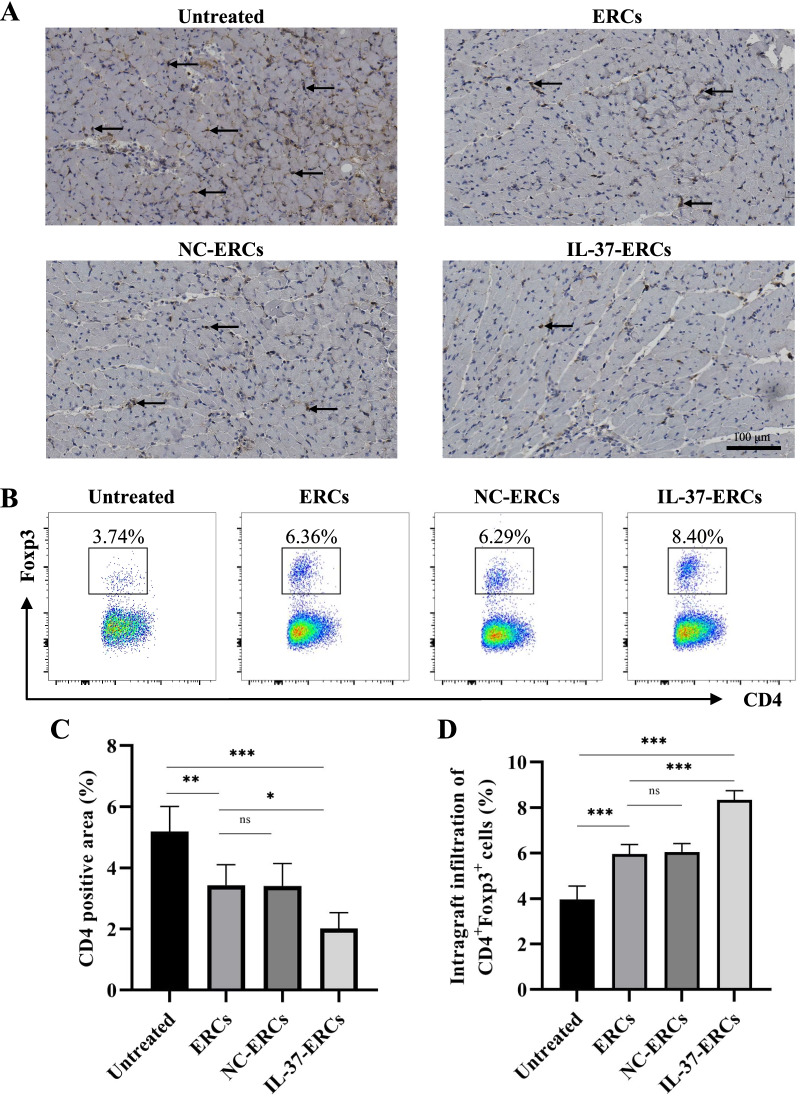
IL-37-ERCs decreased the infiltration of CD4^+^ cells while promoting Treg generation in the graft. **A** Representative graft sections for immunohistological staining of intra-graft CD4^+^ cell infiltration. The positive staining area was shown by an arrow. **B** The mononuclear cells were isolated from the grafts and analyzed by flow cytometry. Treg (CD4^+^Foxp3^+^ cell) populations were significantly raised in the ERC-treated group compared to the untreated and further increased in the IL-37-ERC-treated group (*n* = 6 per group). The percentage of intra-graft CD4^+^ cells (**C**) and Tregs (CD4^+^Foxp3^+^ cells) (**D**) from heart allografts of different groups. Data were analyzed with one-way ANOVA. *P < 0.05, **P < 0.01, ***P < 0.001, ns = non-significant. For all panels, the bar graphs represent mean ± SD

After transplantation, massive inflammatory cells were recruited to the graft, such as CD4^+^ effector T cells, which leads to T cell-mediated acute allograft rejection [47]. To evaluate the effects of IL-37-ERCs on the local immune microenvironment, intra-graft CD4^+^ cell infiltration was detected by immunohistochemistry, and percentage of Tregs (CD4^+^Foxp3^+^) was detected in the grafts by flow cytometry. As shown in Fig. 3, CD4^+^ cell infiltration in ERC-treated group was significantly decreased compared with that of untreated group, and overexpression of IL-37 on ERCs further reduced the infiltration of CD4^+^ cells (Fig. 3A, C, ERC-treated group vs. untreated group, *P* < 0.01; IL-37-ERC-treated group vs. ERC-treated group, *P* < 0.05). Tregs (CD4^+^Foxp3^+^) were remarkably induced in the grafts of ERC-treated group compared with those of untreated group and were further elevated in the IL-37-ERC-treated group (Fig. 3B, D, ERC-treated group vs. untreated group, *P* < 0.001; IL-37-ERC-treated group vs. ERC-treated group, *P* < 0.001). These results indicated that IL-37 overexpression could enhance the ability of ERCs in decreasing CD4^+^ cell infiltration, and increasing Tregs in the grafts following transplantation.

### IL-37-ERCs inhibited the antigen-presenting capacity and stimulatory function of recipients' dendritic cells

**Fig. 4:**
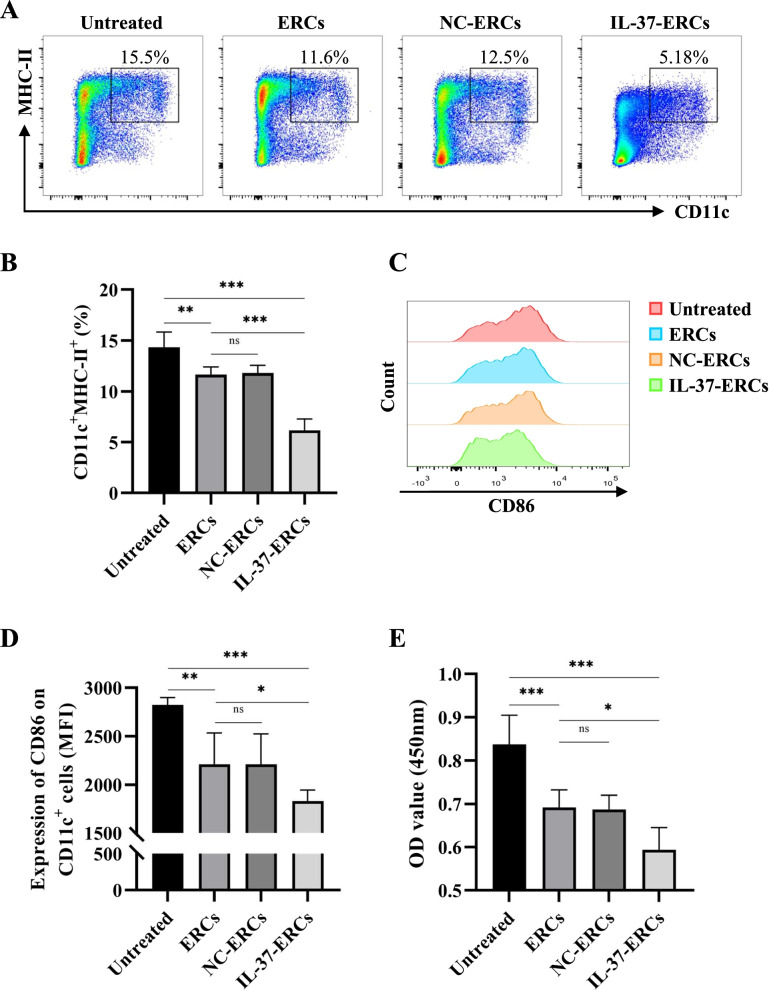
IL-37-ERCs inhibited the antigen-presenting capacity and stimulatory function of recipients' dendritic cells. Single-cell suspensions of splenocytes obtained from the untreated, ERC-treated, NC-ERC-treated, and IL-37-ERC-treated groups were analyzed for the frequency of CD11c^+^MHC-II^+^ cells by flow cytometry, gated on live cells. The representative pseudocolor plots (**A**) and statistical graphs (**B**) were depicted (*n* = 6 per group). In addition, the expression of the costimulatory molecules CD86 was analyzed via flow cytometry. The representative histogram (**C**) and mean fluorescence intensity (MFI) (**D**) are depicted (gated on CD11c^+^ population; *n* = 6 per group). **E** The proliferative response of allogeneic T cells was represented by OD_450_ value, which was measured via the CCK-8 test in one-way MLR analyses (*n* = 6 per group). Statistical analysis was performed using one-way ANOVA, *P < 0.05, **P < 0.01, ***P < 0.001, ns = non-significant. For all panels, the bar graphs represent mean ± SD

Regarded as the most potent antigen-presenting cells (APCs), dendritic cells (DCs) are orchestrators of the immune response, including the acute allograft rejection. So, we next analyzed the effects of IL-37-ERCs on the population and function of splenic DCs using flow cytometry analysis and one‐way MLR. First, we found that the percentage of CD11c^+^MHC-II^+^ cells in ERC-treated group was lower than that of untreated group (ERC-treated group vs. untreated group, *P* < 0.01, Fig. 4A, B) and was further significantly reduced by IL-37-ERC treatment (IL-37-ERC-treated group vs. ERC-treated group, *P* < 0.001; Fig. 4A, B). Costimulatory molecules (CD80, CD86) are necessary for DCs to activate and regulate T cell response. Herein, lower CD86 expression on the CD11c^+^ DC subset was found in ERC-treated group compared with the untreated group (ERC-treated group vs. untreated group, *P* < 0.01, Fig. 4C, D). And further reduction was seen in IL-37-ERC-treated group (IL-37-ERC-treated group vs. ERC-treated group, *P* < 0.05, Fig. 4C, D). Next, we measured the stimulatory capacity of DC to induce T cell proliferation through a one-way MLR. As shown in Fig. 4E, compared with the untreated group, the CD4^+^ T cells proliferation index in ERC-treated group was lower than that of untreated group and was further decreased in the IL-37-ERC-treated group (ERC-treated group vs. untreated group, *P* < 0.001; IL-37-ERC-treated group vs. ERC-treated group, *P* < 0.05). Taken together, IL-37-ERCs inhibited the antigen-presenting capacity and stimulatory function of DCs, which promoted DCs toward a tolerogenic phenotype.

### IL-37-ERCs reduced recipients' splenic Th1 and Th17 populations while increasing the generation of Tregs

**Fig. 5:**
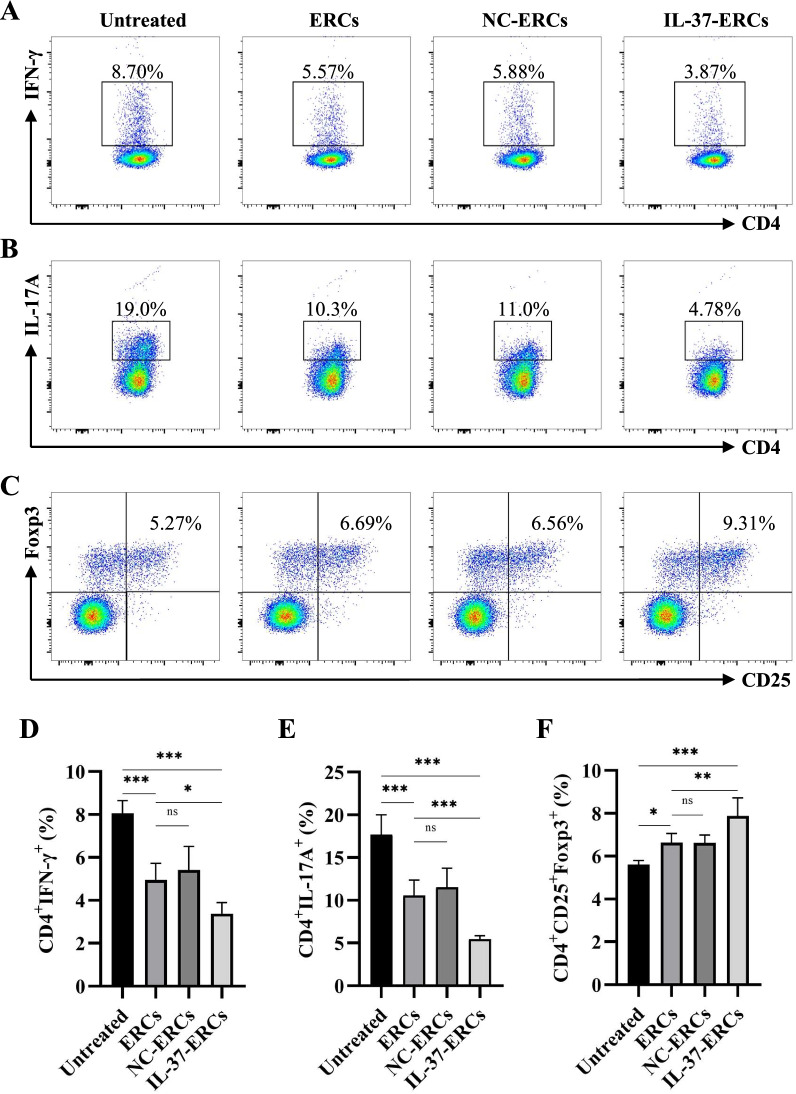
IL-37-ERCs reduced recipients' splenic Th1 and Th17 populations while increasing the generation of Tregs. Splenocytes obtained from the C57BL/6 recipients of each group were stained with CD4^+^IFN-γ^+^IL-17A^+^ and CD4^+^CD25^+^Foxp3^+^, respectively. The representative pseudocolor plots of Th1 cells (**A**), Th17 cells (**B**), and Tregs (**C**) were exhibited. The percentage of **D** Th1 cells, **E** Th17 cells, and **F** Tregs (CD4^+^CD25^+^Foxp3^+^) were analyzed by one-way ANOVA, *n* = 6 per group, *P < 0.05, **P < 0.01, ***P < 0.001, ns = non-significant. For all panels, the bar graphs represent mean ± SD

As mentioned above, CD4^+^ T cells play a pivotal role in acute allograft rejection. Thus, besides the evaluation of DCs, the subset changes of splenic CD4^+^ T cells derived from C57BL/6 recipients were also detected. Splenocytes from different groups were obtained and stained for Th1 (CD4^+^IFN-γ^+^) and Th17 (CD4^+^IL-17A^+^) cells. The proportion of Th1 and Th17 cells were markedly lower in the ERC-treated group when compared with the untreated group (ERC-treated group vs. untreated group, *P* < 0.001), and the proportion was further reduced in the group treated with IL-37-ERCs (Th1: IL-37-ERC-treated group vs. ERC-treated group, *P* < 0.05; Th17: IL-37-ERC-treated group vs. ERC-treated group, *P* < 0.001, Fig. 5A, B, D, E). In addition, as shown in Fig. 5C, a significant upregulation of Tregs (CD4^+^CD25^+^Foxp3^+^) was achieved in the ERC-treated group when compared with the untreated group (ERC-treated group vs. untreated group, *P* < 0.05). Furthermore, IL-37 overexpression (IL-37-ERCs) further enhanced the effect of ERCs (IL-37-ERC-treated group vs. ERC-treated group, *P* < 0.01, Fig. 5C, F). These results suggested that the IL-37 overexpression could promote ERCs to better modulate Th1, Th17, and Treg populations, which in turn attenuate allograft rejection.

### IL-37-ERCs further regulate the serum levels of cytokines in the recipient

**Fig. 6:**
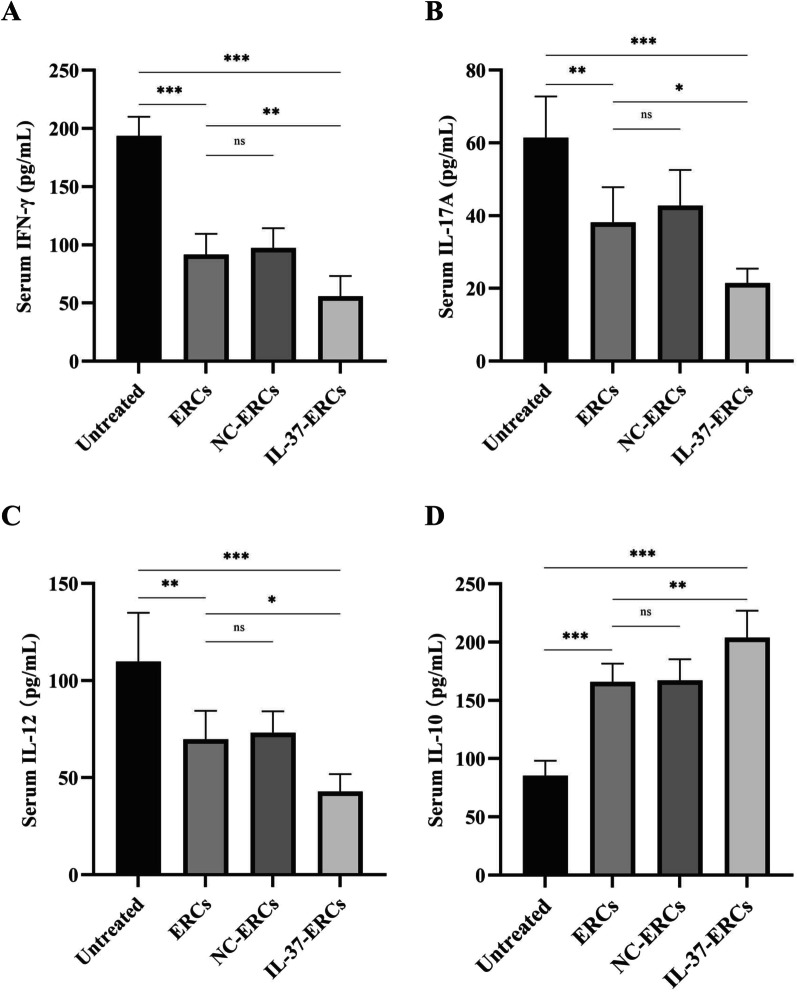
IL-37-ERCs further reduced the levels of pro-inflammatory cytokines but enhanced the level of IL-10 in recipient sera. To evaluate the overall function of the recipients' immune system, the production of pro-inflammatory cytokines and the regulatory cytokine IL-10 in the sera were detected via ELISA assay. The sera were collected from recipients of each group at day 8 post-transplantation. Here, the levels of **A** IFN-γ, **B** IL-17A, **C** IL-12, and **D** IL-10 were shown (*n* = 6 per group). Data were analyzed using one-way ANOVA, *P < 0.05, **P < 0.01, ***P < 0.001, ns = non-significant. For all panels, the bar graphs represent mean ± SD

To detect the levels of inflammatory cytokines in the recipient sera, different ELISA kits were applied. As shown in Fig. 6A–C, the lowest levels of pro-inflammatory cytokines (IFN‐γ, IL-17A, IL-12) were detected in the recipient sera of IL-37-ERC-treated group, while the highest sera levels of those cytokines were found in the untreated group. Compared with the ERC-treated group, IL-37 overexpression significantly promoted ERC-mediated reduction of IFN-γ (IL-37-ERC-treated group vs. ERC-treated group, *P* < 0.01), IL-17A (IL-37-ERC-treated group vs. ERC-treated group, *P* < 0.05), and IL-12 (IL-37-ERC-treated group vs. ERC-treated group, *P* < 0.05). In contrast, IL-10 exhibited the highest level in the sera of IL-37-ERC-treated recipients, while the lowest level of IL-10 was detected in the untreated recipients (Fig. 6D). Meanwhile, the IL-37-ERCs treatment further promoted IL-10 production when compared with ERCs treatment (IL-37-ERC-treated group vs. ERC-treated group, *P* < 0.01). These results revealed that IL-37 overexpression could promote ERC-mediated regulation of cytokines in transplant recipients.

## Discussion

To date, immunosuppressions (including Cyclosporin A, Tacrolimus, Mycophenolate mofetil, etc.) are the predominant therapeutic strategies for inhibition of acute rejection in clinical transplantation [2]. However, the life-long use of immunosuppressions places patients under immunocompromised status and increases the risks of suffering infections and tumors [4, 6]. MSCs have generated intense interest as an innovative cell-based strategy in organ transplantation due to their potential of immunoregulation and low immunogenicity [10, 48]. ERCs have been identified with similar effects to MSCs and can be obtained noninvasively and regularly, expanded easily and quickly, which perfectly meets the requirements of cell therapy.

In the present study, ERCs overexpressing IL-37 were used for preventing acute allograft rejection. Results showed that ERCs alone can prolong graft survival and inhibit acute allograft rejection, which is consistent with our previous studies. Furthermore, we demonstrated that the IL-37 overexpression can promote ERC-mediated therapeutic efficacy in inhibition of acute allograft rejection. Firstly, the IL-37-ERC treatment further prolonged the graft survival compared with ERC treatment. Secondly, the graft rejection index was decreased in either ERC-treated or NC-ERC-treated group and further notably improved in the IL-37-ERC-treated group. Thirdly, IL-37-ERCs promoted the re-establishment of immune balance by regulating CD4^+^ T cells and DCs. Finally, levels of inflammatory cytokines in the sera, which can reflect the systemic inflammatory status, were the highest in the untreated group, and reduced remarkedly after ERCs or NC-ERCs treatment, and further decreased after IL-37-ERC treatment. Taken all together, IL-37 overexpression could promote the therapeutic efficacy of ERCs in inhibition of acute allograft rejection via modulating the immune microenvironment.

DCs are key participants in the development of acute rejection post-allo-transplantation based on their central role in the initiation and regulation of adaptive immune response [49]. Previous studies have indicated that DCs exhibited distinctly different functions depending on their surrounding environmental cues [50, 51]. Predominately, the expression density of MHC-II and costimulatory molecules (CD80, CD86) on cell surface may affect the immune status of DCs, including immune response or tolerance [52]. In the present study, we found that the expression of MHC-II and CD86 was lessened after the cell treatment, especially following IL-37-ERC treatment. Additionally, the serum IL-12, which was mass-produced by the immunogenic DCs, was also declined. Consistent with this, Liu et al. reported that IL-37 inhibited the maturation of DCs in ApoE^−/−^ mice [53]. Lan et al. revealed that recipients' CD11c^+^ DCs obtained from ERC-treated recipients presented a tolerogenic phenotype [31, 33], which express low MHC-II, CD80, CD83, and CD86 and possess poor potential to stimulate allogenic T cells. These data suggested that IL-37 overexpression on ERCs may promote the immunoregulatory efficiency of ERCs through regulating the immunogenesis and tolerogenesis of DCs. IL-37 processed DCs maintained the migratory ability to lymph nodes, but they failed to activate naïve T cells effectively [54]. Using a one-way MLR assay, we found that the CD11c^+^ DCs from the untreated recipients stimulated the highest proliferation index of allogenic CD4^+^ T cells, while IL-37-ERCs treatment demonstrated lowest proliferation index, which revealed that IL-37-ERCs can induce Tol-DCs to promote graft acceptance.

Tregs are a subpopulation of CD4^+^ T cells and maintain acceptance of the allografts in experimental and clinical transplantation by suppressing autoreactive lymphocytes [55–57]. Kushwah et al. revealed that DCs induced tolerance in mice via inducing antigen-specific Treg [58]. Xue et al. showed that DCs transduced with single immunoglobulin IL-1-related receptor (GIGIRR) exhibited immature properties, and thereby upregulating Treg populations in the spleens and lymph nodes, which in turn induces immune tolerance post-islet allografting [59]. Consistent with our previous studies [31, 33, 60], ERCs distinctly increased the percentage of splenic CD4^+^CD25^+^Foxp3^+^ Tregs in the present study. Furthermore, the overexpression of IL-37 can further induce a higher proportion of Tregs in the spleen and the graft. In summary, our present study provides evidence that IL-37 overexpression can promote ERC-mediated therapeutic efficacy by promoting Treg production, thereby ameliorating acute allograft rejection and prolong graft survival.

Converse to Tregs, alloreactive CD4^+^ T cells mediate acute allograft rejection through the recognition of allo-antigens presented by APCs (mainly DCs) [61]. In clinical kidney transplantation, CD4^+^ T cell clones isolated from the patient's renal allografts with acute rejection produced a high level of Th1 cytokines (IFN-γ) after stimulation, indicating that alloreactive Th1 cells are involved in the development of acute rejection [62]. In the present study, Th1 subsets in recipients' splenocytes were markedly downregulated in the ERC-treated group when compared with those of untreated group, and IL-37-ERCs further reduced the Th1 proportion. In line with this, the serum level of IFN-γ, which mediates the immune responses in allograft rejection [63], exhibited the same trends. Besides that, Th17 and its hallmark secretion factor IL-17A have also been remarkedly downregulated in the IL-37-ERC-treated group. IL-17A was first demonstrated to participate in transplant rejection by Van Kooten et al. in 1998 [64]. Since then, many studies have confirmed that Th17 cells and IL-17 take part in acute and/or chronic allograft rejection, especially in acute rejection [65, 66].

The activation and differentiation of naïve CD4^+^ T cells require three signals: antigen presentation on MHC-II, costimulatory molecules, and instructive cell surface and cytokine signals [51]. As the most potent APCs, DCs in conjunction with other accessory cells in the lymph node (LN) microenvironment provide surrounding-dependent third signals, which drive naïve CD4^+^ T cells to differentiate into different subsets [51]. For example, IL-10 promotes Treg production, while IL-12 and IFN-γ promote Th1 differentiation, and IL-17 and IL-22 promote Th17 differentiation [51].

In the present study, some underlying mechanisms need to be discussed. On the one hand, high expression of IL-37 on ERCs conferred successive immune suppression, which prolonged the survival time of ERCs in vivo and, in turn, sustained IL-37 secretion. This positive feedback loop achieves an additive effect of IL-37 and ERCs, as Xu et al. studied in MRL*/lpr* mice for the treatment of SLE [44]. On the other hand, IL-37 overexpression promoted the immunoregulatory efficiency of ERCs, which resulted in the increase in Tol-DCs and Tregs, and the decrease in alloreactive CD4^+^ T cells. However, the specific molecular mechanism of how IL-37-ERCs regulate these immune cells remains to be further studied.

## Conclusion

In conclusion, IL-37 overexpression can promote ERC-mediated inhibition of acute allograft rejection and work as an alternative treatment to minimize the adverse effects of immunosuppressants and prolong allograft survival.

## Data Availability

The dataset supporting the conclusions of this article is included within the article.

## Abbreviations

APCs: Antigen-presenting cells
BM-MSCs: Bone marrow-derived mesenchymal stromal cells
ERCs: Endometrial regenerative cells
GFP: Green fluorescence protein
GIGIRR: Single immunoglobulin IL-1-related receptor
IFN-γ: Interferon-gamma
IL-37: Interleukin-37
IL-37-ERCs: ERCs transfected with IL-37
IL-10: Interleukin-10
IL-12: Interleukin-12
IL-17A: Interleukin-17A
MHC: Major histocompatibility complex
MLR: Mixed lymphocyte reaction
MSCs: Mesenchymal stromal cells
NC-ERCs: ERCs transfected with a vector lacking IL-37 gene
SLE: Systemic lupus erythematosus
TNF-α: Tumor necrosis factor-α
